# Perilipin5 protects against non-alcoholic steatohepatitis by increasing 11-Dodecenoic acid and inhibiting the occurrence of ferroptosis

**DOI:** 10.1186/s12986-023-00751-2

**Published:** 2023-06-22

**Authors:** Xinming Xu, Jin Qiu, Xiaoya Li, Juntong Chen, Yue Li, Xinmei Huang, Shufei Zang, Xinran Ma, Jun Liu

**Affiliations:** 1 Department of Endocrinology, Shanghai Fifth People’s Hospital, Fudan University, 801 Heqing Road, Shanghai, 200240 China; 2 Shanghai Key Laboratory of Regulatory Biological, Institute of Biomedical Science, School of Life Science, East China Normal University, Shanghai, 200241 China

**Keywords:** Perilipin5, Ferroptosis, Steatosis, NASH, 11-Dodecenoic acid

## Abstract

**Background:**

Non-alcoholic steatohepatitis (NASH) is a major contributor to liver cirrhosis and hepatocellular carcinoma. There remains no effective pharmacological therapy. The hepatic lipid metabolism and fatty acid β-oxidation are regulated by Perilipin5 (Plin5). However, it is yet unknown how Plin5 affects NASH and the molecular process.

**Methods:**

High-fat, high-cholesterol and high-fructose (HFHC) diets were used to mimic the progression of NASH in wild type (WT) mice and Plin5 knockout (Plin5 KO) mice. The degree of ferroptosis was measured by detecting the expression of key genes of ferroptosis and the level of lipid peroxide. The degree of NASH was judged by observing the morphology of the liver, detecting the expression of inflammation and fibrosis related genes of liver damage. Plin5 was overexpressed in the liver of mice by tail vein injection of adenovirus, and the process of NASH was simulated by methionine choline deficiency (MCD) diet. The occurrence of ferroptosis and NASH was detected by the same detection method. Targeted lipidomics sequencing was used to detect the difference in free fatty acid expression in the WT Plin5 KO group. Finally, it was verified in cell experiments to further study the effect of free fatty acids on ferroptosis of hepatocytes.

**Results:**

In various NASH models, hepatic Plin5 was dramatically reduced. Plin5 knockout (KO) worsened NASH-associated characteristics in mice given a high-fat/high-cholesterol (HFHC) diet, such as lipid accumulation, inflammation and hepatic fibrosis. It has been shown that ferroptosis is involved in NASH progression. We revealed that Plin5 KO in mice aggravated the degree of ferroptosis in NASH models. Conversely, overexpression of Plin5 significantly alleviated ferroptosis and further ameliorated progression of MCD-induced NASH. Analysis of livers obtained from HFHC diet-fed mice by targeted lipidomics revealed that 11-Dodecenoic acid was significantly decreased in Plin5 KO mice. Addition of 11-Dodecenoia acid to Plin5 knockdown hepatocytes effectively prevented ferroptosis.

**Conclusion:**

Our study demonstrates that Plin5 protects against NASH progression by increasing 11-Dodecenoic acid level and further inhibiting ferroptosis, suggesting that Plin5 has therapeutic potential as a target for the management of NASH.

**Supplementary Information:**

The online version contains supplementary material available at 10.1186/s12986-023-00751-2.

## Background

The prevalence of NAFLD has increased dramatically during the recent past. Metabolic syndrome is a chronic condition that includes several forms of fatty liver. These forms include steatosis, steatohepatitis, and liver fibrosis [1]. NAFLD can progress to NASH, a more serious variant of NAFLD that increases the likelihood of complications such as fibrosis, cirrhosis, and hepatocellular cancer (HCC) [2]. The pathophysiology of NASH is characterized by a number of essential components and markers, including steatosis, hepatocyte destruction, inflammation, and varying degrees of fibrosis [3]. However, no authorized therapeutic pharmacotherapy exists for NASH, and current methods for reducing its consequences fall far short of expectations [4]. This exceedingly frequent condition requires immediate development of a treatment solution.

The energy-storing organelles known as intracellular lipid droplets (LDs) have a neutral lipid core. And it is enriched by a phospholipid monolayer that is made up a large number of proteins. Type 2 diabetes and NASH are two metabolic disorders that are closely linked to the LD accumulation of non-adipose tissue [5]. It is well understood that abnormalities in lipid metabolic pathways results in the accumulation of triacylglycerol (TAG) metabolic intermediates, which causes lipotoxicity and disrupts cellular homeostasis. Importantly, LD accumulation can be coupled or uncoupled by the presence of particular proteins on the LD surface, leading to cellular dysfunction. Perilipins (PLINs) is a protein family composed of five LD proteins and Plin5 is one of them. Plin5 has a high level of expression in the liver, and it can utilize lipids for energy via mitochondrial β-oxidation [6, 7]. Exercise, calorie restriction and fasting all increase Plin5 expression. It has been extensively reported that Plin5 promotes metabolism and relieves LD accumulation caused by insulin resistance and lipotoxicity [8–10]. It Specifically, downstream target gene expression is increased by Plin5. These genes can regulate mitochondrial biogenesis and -oxidation after translocating into the nucleus, where it interacts with and stimulates the activity of SIRT1 and the transcription complex PGC1-α/PPAR-α [11]. However, it is unknown how Plin5 regulates lipid metabolism via other methods.

There is proof that NASH and fibrosis are caused by hepatocyte death [12]. The specific type of iron-dependent cell death known as ferroptosis, which is marked by significant lipid peroxidation, has just lately been discovered. It's different from apoptosis, autophagy, and pyroptosis, which are other types of cell death. Ferroptosis is involved in the advancement of several liver illnesses, including hemochromatosis, alcohol-associated liver disease (ALD), hepatitis C virus (HCV) infection, and hepatocellular carcinoma (HCC), in addition to NASH [13]. NASH patients have been demonstrated to have increased lipid peroxidation, ROS buildup, and liver iron storage [14]. After 24 weeks on a western diet, 10 days of MCD feeding, or a choline-deficient, ethionine-supplemented diet (CDE), ferroptosis was found in these mice [15, 16]. Therefore, targeting ferroptosis could be a possible new therapy method for liver disease patients.

In this research, we found that ferroptosis occurred in HFHC-diet or Methionine- and Choline-deficient (MCD) diet-induced NASH models. Bioinformatic analysis of RNA sequencing datasets of different species of NASH suggest that Plin5 is involved in NASH progression. Plin5 KO in mice aggravated the NASH phenotype and the degree of ferroptosis, while overexpression of Plin5 significantly alleviated ferroptosis and further ameliorated the progression of MCD-induced NASH.

Lipidomic study of WT and Plin5 KO mice fed an HFHC diet liver tissue showed that one type of MUFA, 11-Dodecenoic acid (11-DA), was decreased in the Plin5 KO mice and 11-DA administration rescued ferroptosis-inducing compound RSL-3 treated hepatocytes. This result suggests that Plin5 and downstream MUFA-11-DA may serve as therapeutic targets for inhibiting ferroptosis and NASH progression.

## Methods

### Chemicals and materials

Antibodies against Plin5 (Proteintech,26,951–1-AP), GPX4 (ABclonal, A1933) and GAPDH (Servicebio, GB15004) were purchased commercially. RSL-3 (MedChemExpress, MCE, HY-100218A), Propidium iodide (Sigma, P4170) and 11-Dodecenoic acid (Yuan Ye, B7393) were purchased commercially.

### Absolute quantitative lipidomics and sample preparation

The centrifuge tube was filled with 50 mg of liver tissue, 10 pre-cooled zirconia beads, and 20 μl of deionized water. To extract metabolites, grind for three minutes and then add 120 μl of an internal standard containing methanol. After that, spun at 18000 g for 20 min. Then, transfer the clear liquid remaining after centrifugation to the 96-well plate. The following operations were carried out on a Biomek 4000 workstation (Biomek 4000, Beckman Coulter, Inc., Brea, California, USA). Each well received 40μL of freshly prepared derivative reagent. The plates were sealed and heated to 30 °C for 60 min, then samples were evaporated for 2 h and reconstituted with 330 μl cold 50% methanol solution.

After that, the plates were kept at − 20 °C for 20 min before being centrifuged at 4 °C for 30 min at 4000 g. 135 μl of supernatant was collected in total, and then transferring 10 μl to a new 96-well plate. Internal standards were added to each well. Add the serial diluents of the derived stock standard to the left hole. Finally, the plate seal was utilized for LC–MS analysis.

### Animals and treatments

All animal experiments were carried out in accordance with methods endorsed by East China Normal University's Animal Care and Use Committee. C57BL/6 WT and Plin5 KO mice (8 weeks) were used in the experiment. Plin5 KO mice were bought and generated by Cyagen Biosciences Inc, and created using the CRISPR/ CRISPR-associated 9 (Cas9) system. We used littermate WT mice as control mice. Mice were given a high-fat, high-cholesterol diet (HFHC) (D09100310, Research Diet) diet or normal chow for 24 weeks and were randomly allocated to different experimental groups. The mice were split into two groups: control and AAV-Plin5 group by injection of the adeno-associated virus vector (AAV-vector) and adeno-associated virus Plin5 (AAV-Plin5) respectively. Then mice were fed a Methionine Choline Deficient Diet (MCD) (A02082002BR, Research Diet) or a normal diet for 3 weeks prior to being randomly allocated to different experimental groups.

### Glucose tolerance test (GTT) and Insulin tolerance test (ITT) analysis

GTT and ITT experiments were conducted on mice one week before the end of HFHC feeding. After fasting for 16 h in advance of the GTT, the mice received an injection of glucose (Sigma 47,829) into the intraperitoneal space. Glucose was dissolved in saline at 1.5 g/kg body weight. Insulin was administered intraperitoneally to mice in the ITT experiment at a dose of 0.75 U/kg. Venous blood was obtained from the tail of each mouse 0,15, 30, 60, 90 and 120 min after the injection of glucose or insulin and an ACCU-CHEK Active (Roche) kit used for glucose measurement.

### RNA extraction, quantitative PCR

In accordance with the instructions, RNA was first extracted using Trizol, and then cDNA was generated through reverse transcription using the PrimeScriptTM RT reagent Kit (TaKaRa, RR047A). The RT-PCR experiment was carried out using SYBR green Premix kit (04913914001, Roche) next. Normalize gene expression levels by using β-actin or GAPDH as an internal reference. Additional file 1: Table S1 displays the qPCR primer sequence.

### Western blot analysis

Samples of liver or cells were extracted and quantified. Protein samples were separated on a 10% gel before being transferred to membranes made of polyvinylidene difluoride. Protein expression was observed after incubation overnight at 4 °C with the corresponding primary and secondary antibodies. Quantitative analysis was performed using ImageJ software.

### Mouse liver lipid and function measurements

Using commercial enzymatic kits (Nanjing Jiancheng) and an automatic biochemical analyzer (Roche Cobas C702), serum lipids including triglyceride (TG), total cholesterol (TC) as well as alanine aminotransferase (ALT) and aspartate aminotransferase (AST) were measured. All were evaluated in accordance with the manufacturer's suggested methodology.

### Histological analysis and staining

Mice were sacrificed under 10% chloral hydrate, and liver samples were collected and weighed for further examination. Prussian Blue Iron Staining (Enhance with DAB), Hematoxylin and eosin (H&E), Masson staining and picrosirius red staining (PSR) were applied on paraffin sections. Oil red O staining was applied on frozen liver sections. Three randomly-selected fields were photographed using a Leica DM2500 Microscope.

### NAS scoring

Two experienced pathologists scored the specimens blindly. Hepatic steatosis, lobular inflammation, and hepatocyte ballooning on HE staining were scored separately and then added together to form the NAS score. By combining the scores for steatosis (0–3), lobular inflammation (0–3), and hepatocyte ballooning (0–2), the NAS score—which ranges from 0 to 8—was determined. An NAS score ≥ 5 was strongly associated with a diagnosis of ‘NASH’.

### Iron measurement

The determination of iron and Fe^2+^ in serum and liver tissue should use an iron Assay Kit (Abcam, ab83366). During the test, 10 μl of serum or 30 mg of liver tissue should be used, and then the test should be carried out according to the instructions. Finally, the OD value was measured at 593 nm, and the results were compared with the standard curve of known concentration.

### Fluorescence staining

To conduct the following tests, OCT-embedded frozen liver slices were used. First, treat the liver for 10 min with 4% paraformaldehyde, and then treat it for 10 min with 0.2% Triton X-100 to rupture the liver membrane. Utilize C11-BODIPY (Invitrogen TM D3861,5 Mol/L) for 30 min following PBS washing for three times. The nucleus was then stained with DAPI after another PBS wash. At last, use a confocal microscope to observe and photograph the results.

### Cell culture and siRNA transfection

SK-HEP1, a human liver cancer cell line, was obtained from ATCC and cultured in Media consisting of Dulbecco's modified Eagle's (Gibco) with 10% fetal bovine serum (Gibco) and 50 g/ml penicillin/streptomycin. We constructed Plin5-knockdown hepatocytes by transducing the Plin5 gene with small interfering RNA (siRNA). The siRNA target sequences were as follows: non-targeting control siRNA: sense, 5ʹUUCUCCGAACGUGUCACGUTT-3; antisense, 5ʹACGUGACACGUUCGGAGAATT-3ʹ. hPlin5 siRNA: sense, 5ʹCUGUGGAUGUUGUACUGGATT; antisense, 5ʹUCCAGUACAACAUCCACAGTT-3ʹ. All siRNAs were validated by Western blotting to demonstrate a target knockdown of > 80%. Cell transfection was carried out using GP-transfect-Mate per the manufacturer’s instructions (GenePharma).

### Propidium iodide (PI) staining and cell viability assay

PI was dissolved in PBS at a concentration of 25 μg/ml, then diluted with medium at a ratio of 1:50 and placed in an incubator at 37 °C for 5 min. Use a Leica DM2500 microscope to take photographs.

A Trypan blue staining Cell Viability Assay Kit (Beyotime, C0011) was used to test cell viability. Cells were seeded into 12-well plates and incubated with the indicated treatments. The cells were then collected after trypsin digestion and stained with trypan blue. Cell counting plate was used to determine the number of total live cells and blue cells. Cell viability = (total number of cells − number of blue cells) / total number of cells × 100%.

### MDA and SOD content detection

We used an MDA assay kit and a SOD assay kit (A003-1-1, A001-1-1, Nanjing Jiancheng Bioengineering Institute, China) to measure the amount of MDA and SOD in liver tissue. The level of MDA was measured following the guidelines provided by the manufacturer and TBA method. SOD level was measured following the protocols and hydroxylamine method.

### Cellular lipid ROS assay

Cells were seeded into 6-well dishes at a concentration of 400,000 cells/well. After one days, cells were treated according to group requirements. After treatment, cells were stained with C11-BODIPY 581/591 (Thermo Fisher Scientific, D3861) for 30 min at 37 °C and then harvested by trypsinization. Cells were re-suspended in PBS and then analyzed using flow cytometer (Accuri C6, BD Biosciences) equipped with 488 nm laser for excitation. A minimum of 10,000 cells were analyzed per condition.

### Quantification and statistical analysis

Utilize GraphPad Prism 8.0 to analyze and visualize the experimental data. The figure legend describes the statistical aspects of the experiment. The unpaired Student's *t-test* was used to make statistical comparisons between two groups. *P* < 0.05 was considered statistically significant, *p < 0.05, **p < 0.01.

## Results

### Expression of Plin5 was decreased in NASH models

**Fig. 1:**
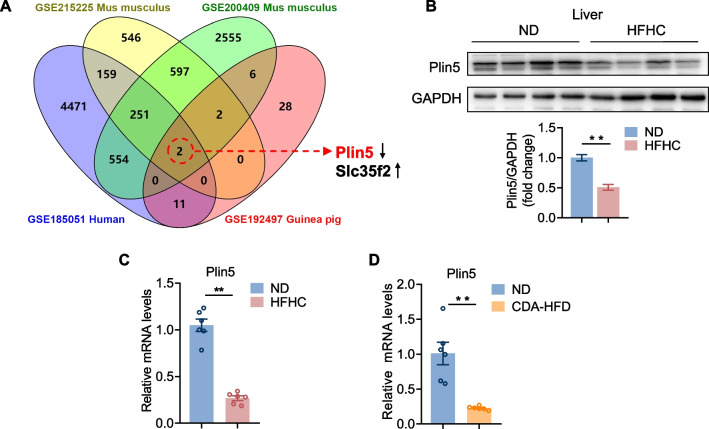
Expression of Plin5 was decreased in fatty liver models. **A** Venn diagram of overlapped genes in NASH group versus control group in the 4 GEO datasets. **B** Representation of western blot and qualification of Plin5 protein level in mice after 24 weeks of ND or HFHC feeding. **C** Quantification of Plin5 mRNA level in mice after 24 weeks of ND or HFHC feeding. **D** qPCR analysis of Plin5 mRNA level in mice under ND or CDA-HFD feeding conditions. A two-tailed Student *t-test* was used for statistical analysis. *< 0.05, **< 0.01. All data are shown as mean ± SEM. (n = 6/group)

To identify molecules important for NASH, we first obtained 4 RNA-seq data for livers with NASH from the GEO database: 1 human (GSE185051), 2 mus musculus (GSE200409, GSE215225) and 1guinea pig (GSE192497). To identify specific genes during NASH, we focused on oscillating genes that featured significant changes (> twofold change and *p* < 0.05) in the NASH group compared with the control group in the four datasets. Results revealed only two genes including one down-regulated gene Plin5 and one up-regulated gene Slc35f2 after overlapping (Fig. 1A). Of note, Plin5 has been shown to be highly abundant in the liver and important for lipid metabolism [17].

To determine whether Plin5 participates in the development of NASH, we identified Plin5 expression changes in two murine models of fatty liver disease. Mice developed serious steatohepatitis after being fed an HFHC diet for 24 weeks or choline-deficient amino acid-defined high fat diet (CDA-HFD) for 12 weeks [18, 19]. Compared with the normal diet (ND) controls, hepatic Plin5 level was dramatically reduced in the liver after feeding an HFHC diet for 24 weeks (Fig. 1B, C) or CDA-HFD for 12 weeks (Fig. 1D). Our data as a whole showed that Plin5 expression was reduced in NASH models.

### Deficiency of Plin5 had no effect on the liver of mice fed a normal chow diet

**Fig. 2:**
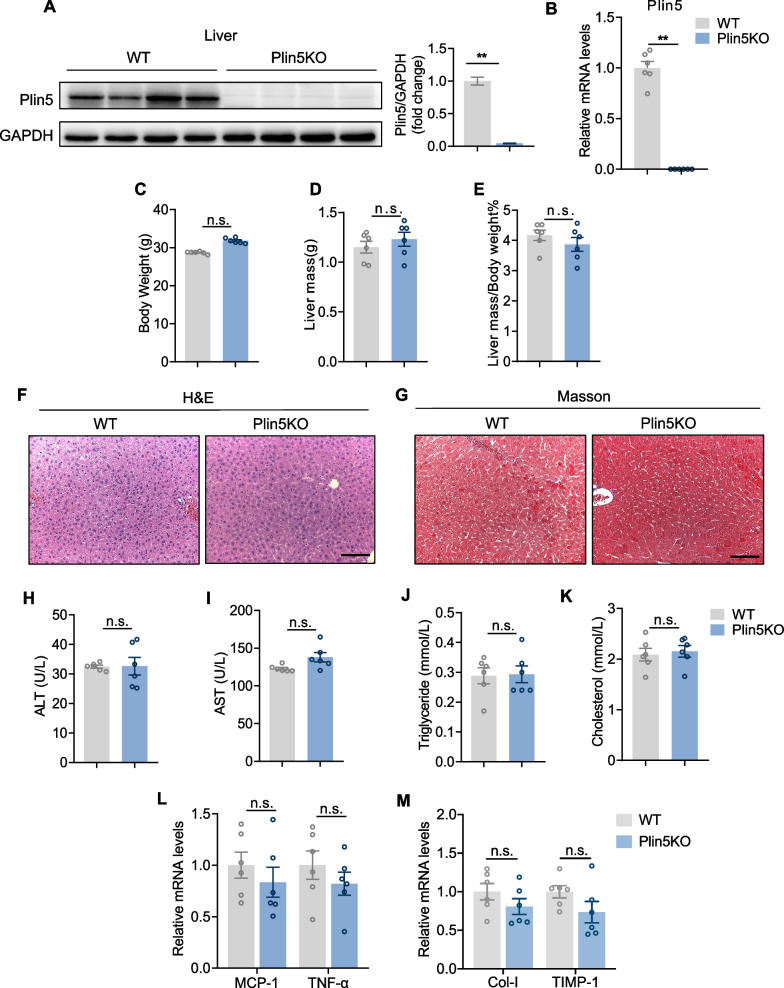
Plin5 deficiency had no effect on liver of mice fed a normal chow diet. **A** Representative western blot and quantification of Plin5 measured in livers of WT and Plin5 KO mice. **B** qPCR analysis of mRNA level of Plin5. **C–E** Body weight (**C**), liver mass (**D**) liver mass/body weight (**E**). **F, G** H&E and Masson staining images of liver sections from WT and Plin5 KO mice. Scale bar, 50 um. **H, I** Serum ALT and AST levels of mice in the indicated groups. **J, K** Serum TG (Triglyceride) and TC (total cholesterol) concentration in WT and Plin5 KO mice. **L** qPCR analysis of mRNA level of inflammation-related genes in the liver of WT and Plin5 KO mice fed a ND diet. **M** Relative mRNA level of profibrotic genes in liver tissue from WT and Plin5KO mice. A two-tailed student t test was used for statistical analysis. *< 0.05, **< 0.01, n.s. not significant. All data are shown as mean ± SEM. Abbreviations: MCP-1: Monocyte Chemoattractant Protein-1; TNF-α: Tumor necrosis factor-α; Collagen α-I: collagen type I alpha1 chain; TIMP-1: TIMP metallopeptidase inhibitor 1. (n = 6/group)

In order to better understand Plin5’s function in the development of NASH, mouse strains with knockout of Plin5 (Plin5 KO) were constructed by CRISPR/Cas-mediated genome engineering. Western blotting and qPCR analysis performed on liver tissue revealed that Plin5 was knocked out in Plin5 KO compared with WT mice (Fig. 2A, B). Plin5 deficiency in mice fed with ND caused a slight increase in body weight but had no effect on liver mass (Fig. 2C–E) or histological appearance (Fig. 2F, G). In addition, serum level of ALT, AST and lipids level were unchanged in Plin5 KO mice fed ND (Fig. 2H–K). Meanwhile, Plin5 deficiency did not influence the expression of inflammatory or collagen related genes in the liver (Fig. 2L, M). These results suggested that Plin5 deficiency had no effect on the liver in mice fed a normal chow diet.

### Plin5 deficiency aggravated HFHC diet-induced steatohepatitis

**Fig. 3:**
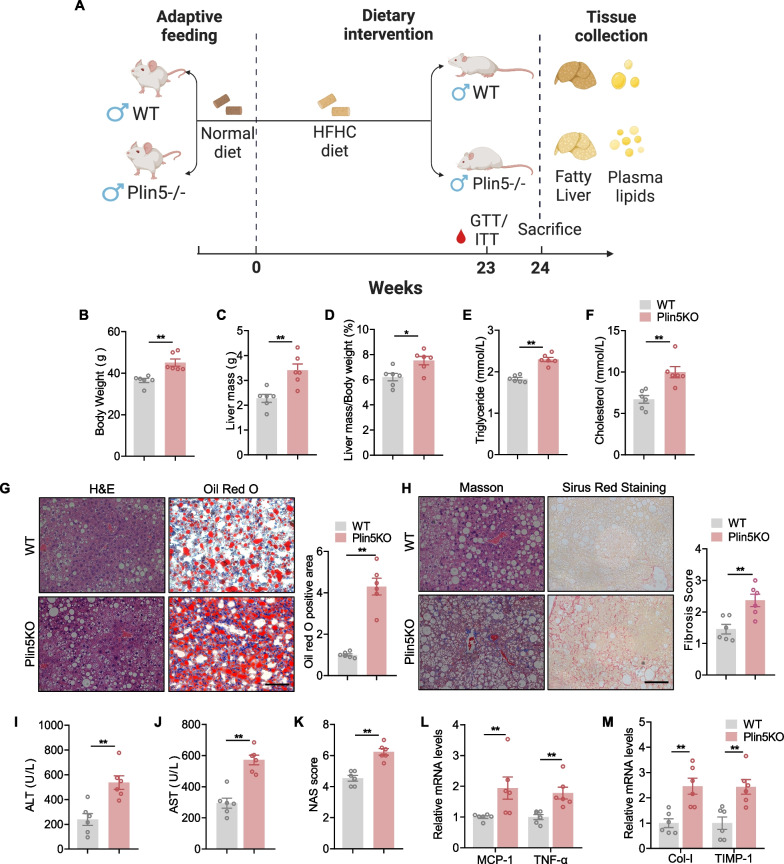
Systemic knockout of Plin5 aggravated HFHC diet-induced steatohepatitis. **A** Schematic diagram of the experimental procedure used to examine the harmful role of Plin5 in mice fed an HFHC for 24 weeks. **B–D** Body weight (**B**), Liver mass (**C**), and liver mass/body weight (**D**) values in WT and Plin5 KO mice after feeding an HFHC diet for 24 weeks. **E, F** Serum TG and TC level. **G** Representative images of H&E and oil red O staining of liver sections from mice fed chow diet or HFHC diet. The statistics of oil red O-positive areas are shown. Scale bar, 50 um. **H** Relative images and quantitative data of PSR staining in liver sections from WT and Plin5 KO mice (scale bars 50 um. **I, J** Serum ALT (**I**) and AST (**J**) were measured in WT and Plin5 KO mice after 24 weeks of HFHC diet feeding (n = 6/group). **K** NAFLD activity score. **L** qPCR analysis of mRNA level of inflammation-related genes in the liver of WT and Plin5 KO mice fed the HFHC diet. **M** Relative mRNA level of profibrotic genes in liver tissue from WT and Plin5 KO mice. A two-tailed Student t test was used for statistical analysis. The data are presented as the mean ± SEM. A significant difference between the WT-ND group and the Plin5 KO-HFHC. group. *< 0.05, **< 0.01; Abbreviations: NAS, NAFLD activity score. (n = 6/group)

To evaluate the potential effects of Plin5 in NASH progression, we examined Plin5 KO mice in an HFHC diet-induced NASH model with induced hepatic steatosis, inflammation and fibrosis, features similar to human NASH. As shown in Fig. 3A, mice fed an HFHC diet or ND were subjected to GTT and ITT, then serum and liver tissue were collected for further analysis. We found that Plin5KO mice had insulin resistance and impaired glucose tolerance (Additional file 1: Fig. S1A, B).

Weight of Plin5 KO mice was significantly higher than that of the WT mice (Fig. 3B) with liver weight and liver weight-to-body weight ratio remarkably increased (Fig. 3C, D). We also observed that Plin5 KO increased TG and TC level in the liver, indicating aggravation of hypertriglyceridemia and hypercholesterolemia by Plin5 deficiency in an HFHC diet-induced NASH mouse model (Fig. 3E, F). Compared with WT mice, Plin5 KO mice showed more significant hepatic fibrosis, as demonstrated by Masson staining and Sirius red staining (Fig. 3H). Meanwhile, Plin5 KO mice fed an HFHC diet showed more severe liver damage, as evidenced by higher serum ALT and AST level (Fig. 3I, J). In addition, NAS score revealed that Plin5 KO aggravated the grade of liver steatohepatitis in the presence of an HFHC-diet (Fig. 3K). NASH is strongly associated with severe inflammation and progressive liver fibrosis [18]. We examined whether Plin5 affected inflammation and fibrosis accompanied by augmented fibrogenic and inflammatory gene expression after feeding an HFHC diet (Fig. 3L, M).

The findings revealed that the HFHC-induced NASH phenotype was worsened by Plin5 KO in hepatocytes.

### Ferroptosis occurs in HFHC-diet fed mouse livers and knockout of Plin5 diminished HFHC diet-induced ferroptosis

Oxidative stress and its associated lipid peroxidation-mediated ferroptosis play key roles in NASH progression [13, 16, 20]. We established that lipid ROS was markedly increased in liver from mice fed an HFHC-diet for 24 weeks, as assessed by specific fluorescent probe C11-BODIPY (581/591) and compared with mice fed an NC diet (Additional file 1: Fig. S2 A). Furthermore, the mRNA levels of genes implicated in ferroptosis, such as Hmox1, Acsl4, Ptgs2, and Nox2, were increased, whereas the protein level of glutathione peroxidase 4 (GPX4), the essential regulator of ferroptosis [21], was lowered (Additional file 1: Fig. S2B, C). These results suggested the occurrence of ferroptosis during NASH progression.

**Fig. 4:**
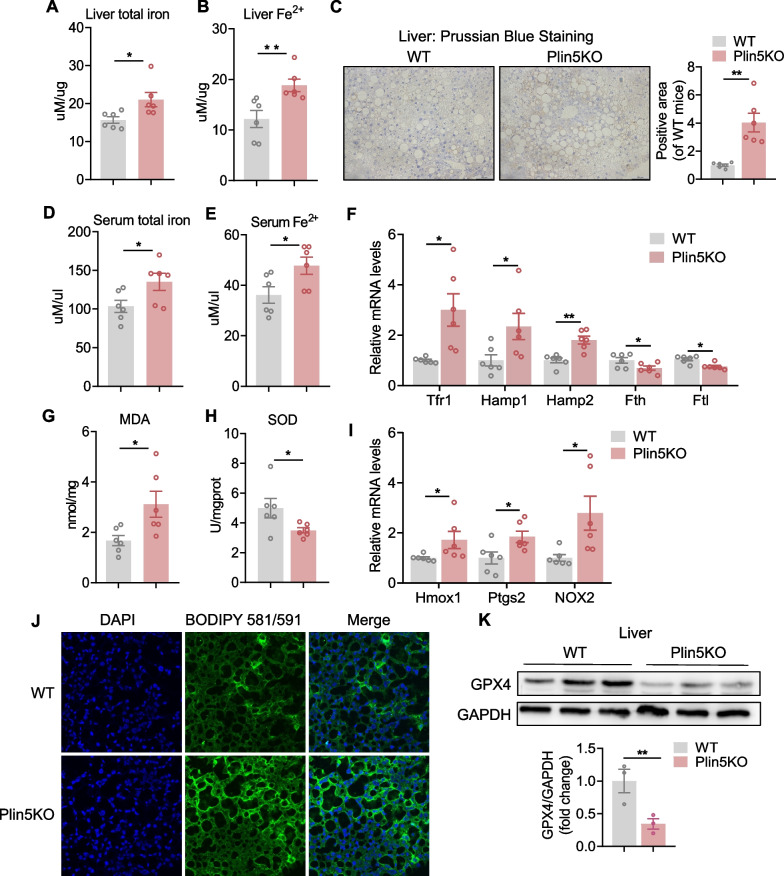
Knockout of Plin5 diminished HFHC diet-induced ferroptosis. **A, B** Total and ferrous iron levels in liver were measured by iron probe in WT and Plin5 KO mice fed an HFHC-diet. **C** Prussian blue iron staining (Enhanced with DAB) of liver sections from mice fed an HFHC-diet. Scale bar, 50 um. **D, E** Serum total and ferrous iron levels were measured by iron probe in WT and Plin5 KO mice fed an HFHC-diet. **F** Expression of hepatic iron metabolic genes, Tfr1, Hamp1, Hamp2, Fth, Ftl, was measured by qPCR from WT and Plin5 KO mice fed an HFHC-diet. **G, H** MDA and SOD content was measured using a malondialdehyde assay kit (TBA method) and total superoxide dismutase assay kit (hydroxylamine method) respectively. **I** Hepatic mRNA level of Hmox1, Ptgs2 and Nox2 was measured by qPCR in HFHC-diet fed mice. **J** Confocal images of WT and Plin5 KO mouse liver sections labeled with C11-BODIPY and DAPI from mice fed on HFHC-diet. Green and blue colors indicate lipid ROS (peroxidated lipids) and nucleus respectively. Scale bar, 50 um. **K** Western blot analysis of GPX4 in WT and Plin5 KO mouse livers. A two-tailed Student *t-test* was used for statistical analysis. The data are presented as the mean ± SEM. *< 0.05, **< 0.01. Abbreviations: MDA: malondialdehyde; SOD: total superoxide dismutase. (n = 6/group)

We further investigated whether lipid droplet Plin5 was involved in the process of iron metabolism and ferroptosis. We first measured total iron and Fe^2+^ level in the liver and serum of WT and Plin5 KO mice. As shown in Fig. 4A, B, Plin5 KO mice showed an increased liver total iron and Fe^2+^ level. Prussian blue staining (Enhance with DAB) also showed increased iron deposits in Plin5 KO mouse liver (Fig. 4C). Meanwhile, Plin5 KO mice demonstrated increased serum total iron and Fe^2+^ level (Fig. 4D, E). Iron metabolism-related genes including Hamp1, Hamp2 and iron transport gene Tfr1 were significantly increased, while iron storage related genes Ferritin H heavy chain (Fth) and light chain (Ftl) were significantly decreased in Plin5 KO mouse liver compared with that of WT mice (Fig. 4F). Furthermore, increased MDA and diminished SOD content were evident in the liver of Plin5 KO mice (Fig. 4G, H).

In addition, the positive regulators of ferroptosis Hmox1, Ptgs2 and NOX2 were significantly up-regulated in Plin5 KO mice (Fig. 4I). The occurrence of lipid peroxidation in the liver was further confirmed by markedly increased lipid ROS production (Fig. 4J). Nonetheless the expression of GPX4, which is the negative regulator of ferroptosis, was decreased in Plin5 KO mice (Fig. 4K). These results suggested that deletion of Plin5 aggravated the occurrence of ferroptosis in an HFHC-induced mouse model.

### Overexpression of Plin5 ameliorated MCD diet-induced NASH and ferroptosis

**Fig. 5:**
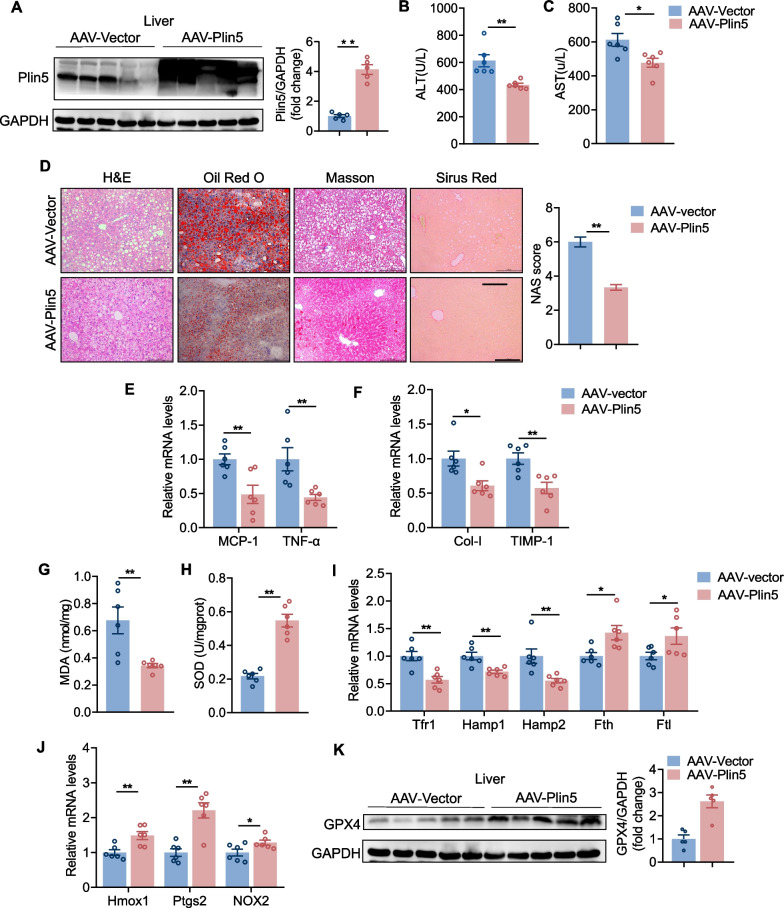
Hepatocyte-specific overexpression of Plin5 alleviated MCD-induced NASH and ferroptosis. **A** Representative western blot of Plin5 measured in livers of AAV-vector and AAV-Plin5 mice. **B, C** Serum level of ALT and AST measured in WT and Plin5 KO mice after 3 weeks of MCD feeding. **D** Representative images of H&E, oil red O staining, Masson staining and PSR staining of liver sections from mice and NAFLD activity scores (Scale bar,100 um). **E, F** qPCR analysis of mRNA level of inflammation-related genes and profibrotic genes in the liver of AAV-vector and AAV-Plin5 mice fed an HFHC diet. **G, H** MDA and SOD content were measured by malondialdehyde assay kit (TBA method) and total superoxide dismutase assay kit (hydroxylamine method) respectively. **I** Expression of hepatic iron metabolic genes Tfr1, Hamp1, Hamp2, Fth and Ftl were measured by qPCR from AAV-vector and AAV-Plin5 mice under MCD-diet condition. **J** Hepatic mRNA level of Hmox1, Ptgs2, and Nox2 were measured by qPCR in MCD-diet mice. **K** The protein level of GPX4 and GAPDH in AAV-vector and AAV-Plin5 mice fed an MCD-diet were detected by western blot. A two-tailed Student *t-test* was used for statistical analysis. Data are presented as the mean ± SEM. *< 0.05, **< 0.01. (n = 6/group)

To further validate the role of Plin5 in steatohepatitis, we administered AAV-Plin5 or AAV-Vector to mice and fed them an MCD for 3 weeks to induce steatohepatitis [22]. Western blot experiments showed that the expression of Plin5 was significantly increased in liver tissue from AAV-Plin5 mice compared with the control group (Fig. 5A). Compared with MCD fed WT mice, serum levels of hepatic enzymes (AST and ALT) were significantly reduced in AAV-Plin5 mice, indicating that Plin5 significantly ameliorated liver injury (Fig. 5B, C). Furthermore, histological staining and the grade of steatohepatitis determined by NAS score demonstrated a reduction in hepatic steatosis and fibrosis for MCD-diet fed AAV-Plin5 mice (Fig. 5D) accompanied by reduced inflammatory and fibrosis-related genes (Fig. 5E, F).

Meanwhile, the reduction of MDA and excess of SOD were significantly improved after Plin5 over-expression (Fig. 5G, H). Hepatic iron metabolism genes including Hamp1, Hamp2, and Tfr1 were significantly down-regulated, while Fth and Ftl were significantly up-regulated in the livers of the AAV-Plin5 group (Fig. 5I). We also found that the Hmox1, Ptgs2 and NOX2 were decreased and protein expression of GPX4 was increased in AAV-Plin5 mice, indicating that Plin5 over-expression effectively reduced ferroptosis in MCD-induced NASH (Fig. 5J, K). These data demonstrate that overexpression of Plin5 inhibited ferroptosis and alleviated NASH progression.

### Plin5 KO aggravated RSL-3-induced ferroptosis and could be reversed by 11-Dodecenoic acid, a downstream metabolite of Plin5

Exogenous MUFAs have been shown to prevent ferroptosis in both transformed and non-transformed cells through a structure-specific mechanism [23]. Exogenous monounsaturated fatty acids inhibit ferroptosis, a non-apoptotic mechanism, via increasing ACSL3-dependent polyunsaturated fatty acid substitution from plasma membrane phospholipids [24].

**Fig. 6:**
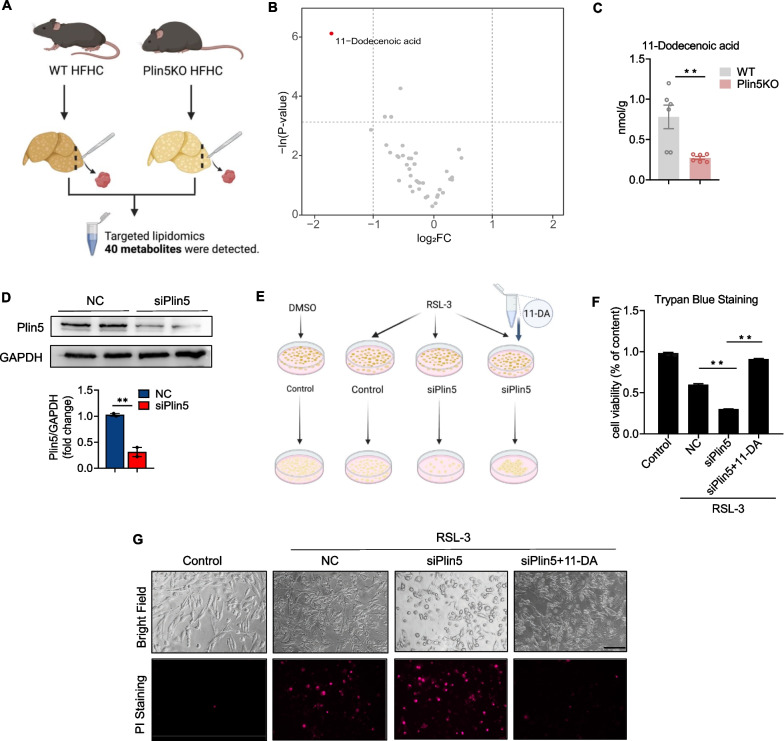
Plin5 KO aggravated RSL-3 induced ferroptosis that was reversed by 11-Dodecenoic acid, a downstream targeting molecular of Plin5. **A** Schematic of the experimental strategy used to identify the potential target metabolites of Plin5. in steatotic hepatocytes. **B** Volcano plot indicates different metabolites between the Plin5 KO group and the control group. **C** Concentration of 11-Dodecenoic acid in WT and KO groups. **D** Protein expression and quantification of Plin5 in SK-HEP1 cells infected with siPlin5. **E** Schematic diagram of cells experiment. **F** Cell viability was assayed by trypan blue staining. **G** SK-HEP1 cells with empty vector, Plin5 knockdown, and adding 11-DA were treated with RSL-3 for 12 h and cell states were captured by microscope. Dead cells were stained by propidium iodide (PI). Scale bar, 100 um

Since Plin5 is involved in lipid metabolism, we explored the possible downstream metabolite by which Plin5 affected NASH and ferroptosis via targeted lipidomics with liver tissue from Plin5 KO mice and WT mice fed an HFHC-diet (Fig. 6A). A total of 40 metabolites were detected: 11 types of MUFAs, 3 types of TUFAs, 14 types of PUFAs and 12 types of SFAs. Interestingly, volcano plot revealed that compared with the WT group, 11-Dodecenoic acid (11-DA) in liver was significantly down-regulated (*p* < 0.05 and |log2FC|≥ 1) in the Plin5 KO group under HFHC diet conditions (Fig. 6B, C), suggesting that 11-DA may be an important metabolite downstream of Plin5 during NASH progression. In addition, the total amount of MUFAs and PUFAs was not significantly altered in Plin5KO group compared with WT group (Additional file 1: Fig. S3A, B).

To examine the functional roles of Plin5 in hepatocytes, we created Plin5-knockdown hepatocytes by transducing the Plin5 gene with a short interfering RNA (siRNA). Western blot analysis revealed that siRNA (siPlin5) significantly inhibited Plin5 expression in the siPlin5 group relative to the negative control (NC) group (Fig. 6D). Hepatocyte cell line SK-HEP1 is frequently utilized for ferroptosis research because to its extreme sensitivity to ferroptosis-inducing chemicals such the GPX4 inhibitor RSL-3, which causes lipid peroxidation and ferroptosis when present in sufficient amounts [25]. We treated control or siPlin5 hepatocytes with or without 11-Dodecenoic acid following RSL-3 administration (Fig. 6E). Of note, siPlin5 hepatocytes demonstrated significantly increased RSL-3-induced ferroptosis and cell death while 11-DA (200 μM) rescued the effects (Fig. 6F, G).

Excessive accumulation of lipid peroxide (lipid ROS), generated by the family of lipoxygenases, is a critical cause leading to ferroptosis [26]. Lipid ROS can be detected by using fluorescent radio-probe C11-BODIPY 581/591. We examined lipid ROS expression levels in different groups. It was found that under RSL3 treatment conditions, the expression of lipid ROS was increased after knocking down Plin5, while the level of lipid ROS was decreased after adding 11-DA (Additional file 1: Fig. S3C).

We concluded that Plin5 knockdown in hepatocytes aggravated RSL-3-induced ferroptosis, while addition of 11-DA rescued the phenotype, suggesting that 11-DA may be a downstream metabolite effector of Plin5.

## Discussion

Metabolic syndrome, which typically comprises obesity, diabetes, and dyslipidemia, manifests in the liver as nonalcoholic fatty liver disease (NAFLD) [27]. There is strong evidence that altered hepatic and extrahepatic lipid metabolism are the main drivers [28, 29].

The liver plays a significant role in lipid metabolism. The interaction of hormone level, lipoproteins, transcription factors and nuclear receptor take part in controlling the lipid level in the liver [30]. The accumulation of fat in the liver and the emergence of NAFLD may come from the destruction of one or more of these mechanisms. Inconsistency between lipid disposal and absorption causes hepatic fat accumulation, which is regulated by four major pathways: de novo lipogenesis (DNL), fatty acid oxidation (FAO), uptake of circulating lipids, and export of lipids in very low-density lipoproteins (VLDL).

PLINs are known as representation of LDs and necessarily associated with LDs formation. Plin5 is highly expressed in oxidative tissues and generally restricted to tissues/cells that utilize lipids for energy mitochondrial β-oxidation [31, 32]. Previous studies have reported the involvement of Plin5 in hepatic lipid metabolism. A study shows that cells overexpressing Plin5 release lower amounts of FFAs in basal conditions [33]. In Plin5 deficient mice, TG content was found to decrease both in primary mouse hepatocytes and in the liver, implying the involvement of Plin5 in TG accumulation [34]. These findings suggest that Plin5 can prevent the FFA and its metabolites over accumulation, thus preventing liver toxicity damage. Therefore, Plin5 is involved in the process of lipid metabolism in hepatic tissues.

Plin5 is uncoupled from LD accumulation by lipotoxicity and metabolic dysfunction, and it is favorably linked with triglyceride storage and fatty acid oxidation. Plin5 promotes triglyceride hydrolysis and fatty acid oxidation in response to lipolysis stimulation, such as cAMP/PKA signaling, but normally interacts with and inhibits ATGL. It has been established in recent studies that Plin5 promotes PGC-1α/PPAR-α activity by interacting with PGC-1α and SIRT1 [35, 36]. Overall, Plin5 influences lipid metabolism and NASH, but the exact method by which it does so is not entirely understood.

Ferroptosis is involved in the development of NASH, according to earlier investigations. An expanding number of metabolic routes have been discovered to be connected to ferroptosis in addition to the iron metabolism in the condition, such as the cysteine/glutathione (GSH)/GPX4 axis, the guanosine triphosphate cyclohydrolase 1(GCH1)/tetrahydrobiopterin (BH4)/dihydrofolate reductase (DHFR) axis, and the ferroptosis suppressor protein 1 [37]. Abnormal accumulation of lipid in the liver is also a hallmark of NAFLD. One of the hallmarks of ferroptosis is an abnormal buildup of lipid peroxidation [38]. Thus,.in the present study, we have shown that Plin5 protects against NASH by increasing 11-Dodecenoic acid and inhibiting the occurrence of ferroptosis, thus linking lipid metabolic regulation and ferroptosis in the NASH progression.

One of the characteristics of ferroptosis is the increase of lipid peroxidation [38]. Exogenous monounsaturated fatty acids (MUFAs) have recently been discovered to be the cell-intrinsic method of hiding PUFAs from plasma membrane phospholipids, thus preventing lipid peroxidation. In addition, the biosynthesis of MUFA from saturated fatty acids requires stearoyl-CoA desaturase (SCD) [24, 38]. Overexpressed SCD1 in ovarian cancer cells prevented ferroptosis, simulating the effects of MUFA therapy [39]. Cell death by ferroptosis requires insufficient regulation of intracellular iron accumulation and PUFA-enriched phospholipids. Thus, MUFAs exert anti-ferroptotic effects because they compete with PUFAs for phospholipids incorporation. 11-DA is a type of monounsaturated fatty acid (MUFA) that can bind to Plin5 and activate its downstream target gene SIRT1 [11]. MUFAs are key components of the Mediterranean diet that has been recognized to have broad health benefits. Furthermore, nuts, avocados, olive oil and many other foods are rich in MUFAs. There is evidence from model biology research and clinical trials that MUFAs can increase energy expenditure and oxidative metabolism [11, 40, 41].

Previous research has discovered that MUFAs are endogenous non-substrate regulators of SIRT1 and that they can direct deacetylase to certain protein substrates. PKA-mediated phosphorylation is a critical event that improves Plin5's binding capacity to MUFAs and causes it to translocate to the nucleus. These findings also verified Plin5's new role in fatty acid binding and transport, which could be a mechanism for linking lipolysis to SIRT1/PGC-1a signaling [7, 40, 42]. Despite the fact that we employed systemic knockout mice, our Plin5 overexpression animals and cell assays at least partially provided evidence that hepatocyte Plin5 regulates ferroptosis and NASH. Similarly, Plin5 may influence many distinct downstream targets and play a crucial role in the development of NAFLD. Nonetheless the mechanisms that underlie regulation of lipid turnover by Plin5 are still unclear. The potential contribution and effect of 11-Dodecenoic acid (MUFAs) on Plin5 warrants further study.

It is well known that Plin5 is highly expressed in oxidative tissues and may share different functions in different tissues [43]. Recent finding suggests Plin5 interacted with PGC-1α and affecting the generation of ROS. Knockdown of Plin5 attenuated its interaction with PGC-1α, accompanied by increased ROS levels [44]. Furthermore, another studies identified the first-known endogenous allosteric modulators of SIRT1 and characterize a LD-nuclear signaling axis that underlies the known metabolic benefits of MUFAs and Plin5 [45]. What’s more, Wang et al. found that Plin5 localized on the LD and regulated the interaction between ATGL and CGI-58 [36]. Based on the literature and previous studies, we hypothesized that Plin5 may play a role in ferroptosis by interacting with PGC-1α, SIRT1 or CGI-58 to affect 11-DA or other lipid production. The above speculation and the specific interaction mechanism will be verified in our future experiments.

In summary, our results revealed the role of Plin5 in the prevention of NASH by increasing 11-DA and inhibiting ferroptosis and revealed a target for NASH therapy.

## Supplementary Information

**Additional file 1: Table S1**. Sequences of primers used for qPCR. **Fig. S1. **Glucose metabolism in WT and Plin5KO mice fed a HFHC diet. **A** Intraperitoneal GTT. **B** Intraperitoneal insulin tolerance test. **Fig. S2.** HFHC diet treatment induced ferroptosis in mouse livers. **A** Confocal images of liver sections labeled with C11-BODIPY and DAPI from mice fed on ND or HFHC diet. Green and blue colors indicate lipid ROSand nucleus respectively. **B** Hepatic mRNA levels of Hmox1, Acsl4, Ptgs2, NOX2 were measured by RT-PCR in ND and HFHC-diet fed mice. **C** Western blot showing expression levels of GPX4 in the indicated group, GAPDH served as a loading control. Summary data are presented as the mean ± SEM. *<0.05, **<0.01. Abbreviations: Tfr1: transferrin receptor; Hamp1: Hepcidin Antimicrobial Peptide1; Hamp2: Hepcidin Antimicrobial Peptide2; Fth: Ferritin heavy chain; Ftl: ferritin light chain.. **Fig. S3.** **A**, **B** The total levels of MUFAs and PUFAs in the liver tissues of WT and Plin5KO groups. **C** Assessment of lipid ROS accumulation by C11-BODIPY 581/591 staining coupled with flow cytometry analysis.

## Data Availability

This article contains supplemental data. All data are provided within the main text and supplemental document.

## Abbreviations

MCP-1: Monocyte chemoattractant protein-1
TNF-α: Tumor necrosis factor-α
Collagen α-I: Collagen type I alpha1 chain
TIMP-1: TIMP metallopeptidase inhibitor 1
NAS: NAFLD activity score
MDA: Malondialdehyde
SOD: Total superoxide dismutase
11-DA: 11-Dodecenoic acid
Tfr1: Transferrin receptor
Hamp1: Hepcidin antimicrobial peptide1
Hamp2: Hepcidin antimicrobial peptide2
Fth: Ferritin heavy chain
Ftl: Ferritin light chain
