# Supplementary material for: Perilipin5 protects against non-alcoholic steatohepatitis by increasing 11-Dodecenoic acid and inhibiting the occurrence of ferroptosis
Source: Nutr Metab (Lond). 2023 Jun 22;20:29. doi: 10.1186/s12986-023-00751-2 (PMC10286369; doi:10.1186/s12986-023-00751-2)
Supplement: Supplementary file 1 — Additional file 1: Table S1. Sequences of primers used for qPCR. Fig. S1. Glucose metabolism in WT and Plin5KO mice fed a HFHC diet. A Intraperitoneal GTT. B Intraperitoneal insulin tolerance test. Fig. S2. HFHC diet treatment induced ferroptosis in mouse livers. A Confocal images of liver sections labeled with C11-BODIPY and DAPI from mice fed on ND or HFHC diet. Green and blue colors indicate lipid ROSand nucleus respectively. B Hepatic mRNA levels of Hmox1, Acsl4, Ptgs2, NOX2 were measured by RT-PCR in ND and HFHC-diet fed mice. C Western blot showing expression levels of GPX4 in the indicated group, GAPDH served as a loading control. Summary data are presented as the mean ± SEM. *<0.05, **<0.01. Abbreviations: Tfr1: transferrin receptor; Hamp1: Hepcidin Antimicrobial Peptide1; Hamp2: Hepcidin Antimicrobial Peptide2; Fth: Ferritin heavy chain; Ftl: ferritin light chain.. Fig. S3. A, B The total levels of MUFAs and PUFAs in the liver tissues of WT and Plin5KO groups. C Assessment of lipid ROS accumulation by C11-BODIPY 581/591 staining coupled with flow cytometry analysis. [file 12986_2023_751_MOESM1_ESM.docx]

**Table S1.** Sequences of primers used for qPCR.

| **Primer** | **Sequences （5' to 3'）** |
| --- | --- |
| mTimp1-F | TGAGCCCTGCTCAGCAAAGA |
| mTimp1-R | GAGGACCTGATCCGTCCACAA |
| mCollagen I-F | ACCTGTGTGTTCCCTACTCA |
| mCollagen I-R | GACTGTTGCCTTCGCCTCTG |
| mGAPDH-F | CTCATGACCACAGTCCATGC |
| mGAPDH-R | CACATTGGGGGTAGGAACAC |
| mβ-actin-F | ATGACCCAGATCATGTTTGA |
| mβ-actin-R | TACGACCAGAGGCATACAG |
| mMCP1-F | TCCCAATGAGTAGGCTGGAG |
| mMCP1-R | TCTGGACCCATTCCTTCTTG |
| mTNFα-F | CCAGGCGGTGCCTATGTCTC |
| mTNFα-R | CAGCCACTCCAGCTGCTCCT |
| mPlin5-F | TGTCCAGTGCTTACAACTCGG |
| mPlin5-R | CAGGGCACAGGTAGTCACAC |
| mHmox1-F | AGGTACACATCCAAGCCGAGA |
| mHmox1-R | CATCACCAGCTTAAAGCCTTCT |
| mAcsl4-F | CCTGAGGGGCTTGAAATTCAC |
| mAcsl4-R | GTTGGTCTACTTGGAGGAACG |
| mPtgs2-F | TTCAACACACTCTATCACTGGC |
| mPtgs2-R | AGAAGCGTTTGCGGTACTCAT |
| mNox2-F | AGTGCGTGTTGCTCGACAA |
| mNox2-R | GCGGTGTGCAGTGCTATCAT |
| mTfr1-F | CTCAGTTTCCGCCATCTCAGT |
| mTfr1-R | GCAGCTCTTGAGATTGTTTGCA |
| mHamp1-F | GCACCACCTATCTCCATCAACA |
| mHamp1-R | TTCTTCCCCGTGCAAAGG |
| mHamp2-F | ACACAAGTCCTTAGACTGCACA |
| mHamp2-R | TTCTGCATTGGGATCGCAAT |
| mFth-F | CCATCAACCGCCAGATCAAC |
| mFth-R | GAAACATCATCTCGGTCAAA |
| mFtl-F | CGTCTCCTCGAGTTTCAGAAC |
| mFtl-R | CTCCTGGGTTTTACCCCATTC |


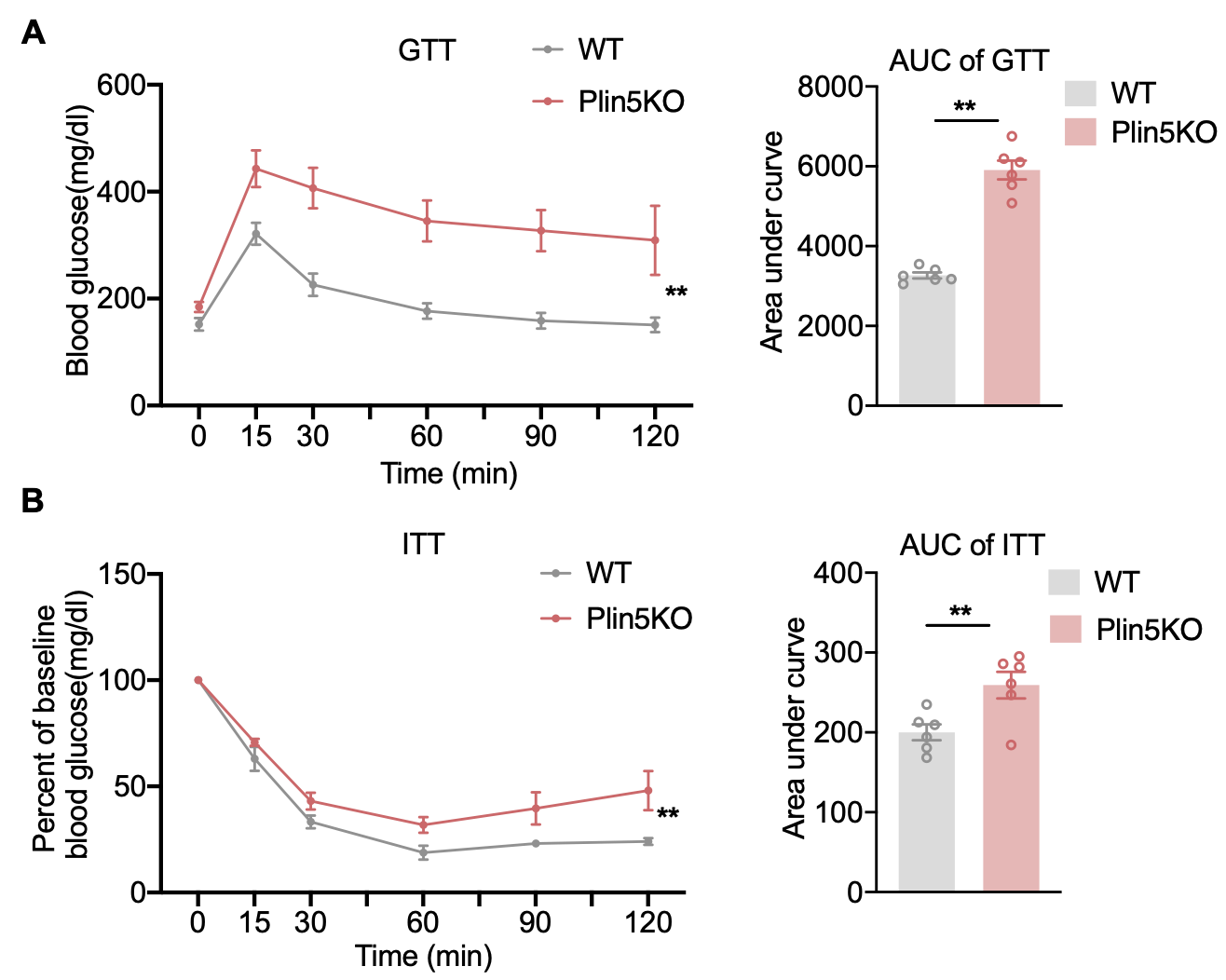


**Figure S1.** Glucose metabolism in WT and Plin5KO mice fed a HFHC diet. (**A**) Intraperitoneal GTT (1.5g/kg; n=6/group). (**B**) Intraperitoneal insulin tolerance test (0.75 U/kg; n=6/group).


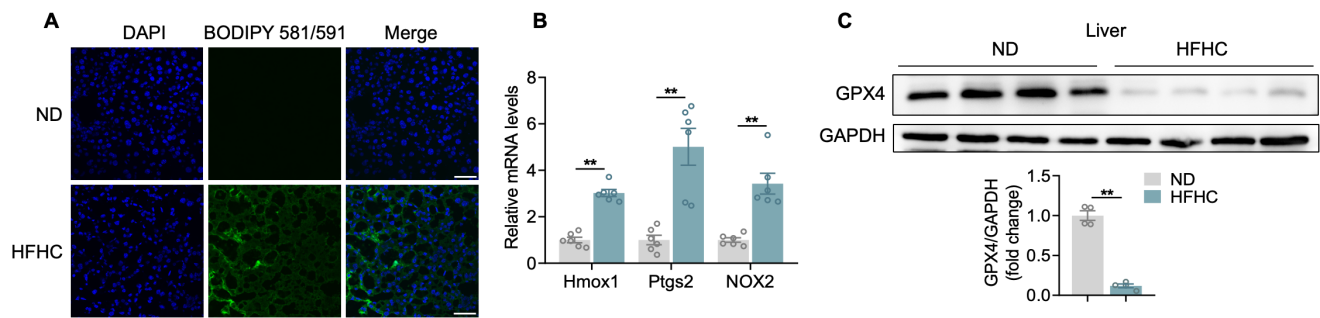


**Figure S2.** HFHC diet treatment induced ferroptosis in mouse livers. (**A**) Confocal images of liver sections labeled with C11-BODIPY and DAPI from mice fed on ND or HFHC diet. Green and blue colors indicate lipid ROS (peroxidated lipids) and nucleus respectively. (**B**) Hepatic mRNA levels of Hmox1, Acsl4, Ptgs2, NOX2 were measured by RT-PCR in ND and HFHC-diet fed mice (n=6/group). (**C**) Western blot showing expression levels of GPX4 in the indicated group, GAPDH served as a loading control. Summary data are presented as the mean ± SEM. *<0.05, **<0.01. Abbreviations: Tfr1: transferrin receptor; Hamp1: Hepcidin Antimicrobial Peptide1; Hamp2: Hepcidin Antimicrobial Peptide2; Fth: Ferritin heavy chain; Ftl: ferritin light chain.

**
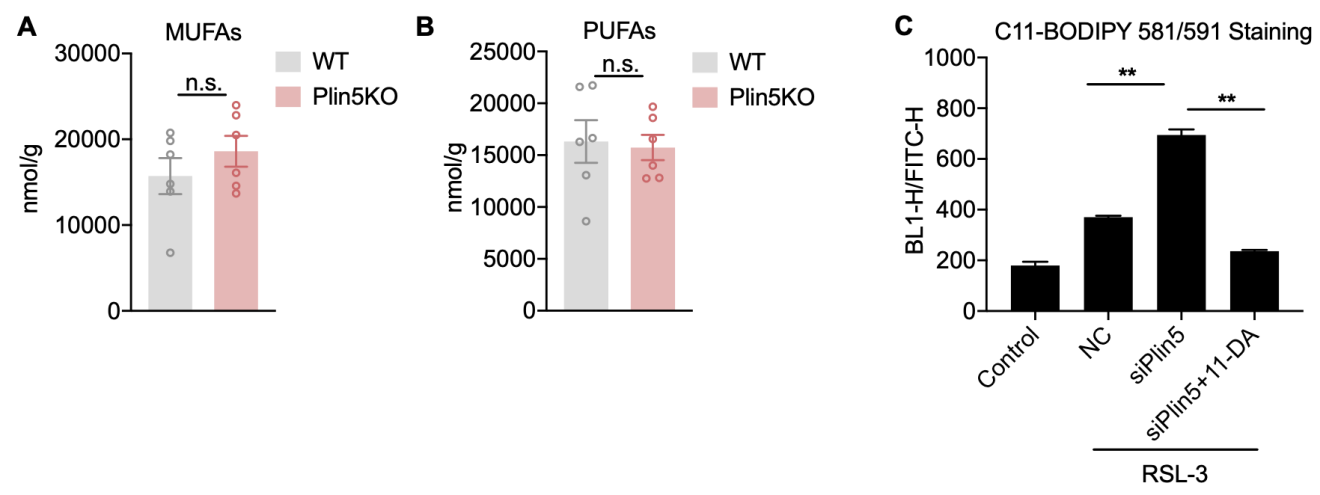
**

**Figure S3.** (**A**, **B**) The total levels of MUFAs and PUFAs in the liver tissues of WT and Plin5KO groups (n=6/group). (**C**) Assessment of lipid ROS accumulation by C11-BODIPY 581/591 staining coupled with flow cytometry analysis (n=3/group).
